# Optic Neuropathy Associated with Recreational Abuse of Nitrous Oxide: A Retrospective Case Series

**DOI:** 10.3390/jcm15197475

**Published:** 2026-09-25

**Authors:** Joseph Albou, Julien Fallouh, Bertrand Degos, Franck Fajnkuchen, Sylvia Nghiem-Buffet, Julie Mazoyer, Audrey Giocanti-Aurégan, Héloïse Torres-Villaros

**Affiliations:** 1 Ophthalmology Department, Avicenne Hospital, 125 rue de Stalingrad, 93000 Bobigny, France; jalbou@15-20.fr (J.A.); buffet.nghiem@free.fr (S.N.-B.); audrey.giocanti@aphp.fr (A.G.-A.); 2 Neurology Department, Avicenne Hospital, 93009 Bobigny, France; bertrand.degos@aphp.fr (B.D.);; 3 Sorbonne Paris Nord University, 93000 Bobigny, France; 4 Centre d’Imagerie et de Laser, 75015 Paris, France

**Keywords:** nitrous oxide, laughing gas, optic neuropathy, vitamin B12

## Abstract

**Background/Objectives**: Nitrous oxide is known to cause neurological damage. We investigated the potential effects of nitrous oxide (N_2_O) abuse on the optic nerve through comprehensive ophthalmological evaluation of patients diagnosed with N_2_O-related neurological disorders. **Methods**: This retrospective, monocentric case series included patients hospitalized for N_2_O-related neurological manifestations in the neurology department of a French tertiary care center. Suspected optic neuropathy was defined as abnormal retinal nerve fiber layer (RNFL) and ganglion cell layer (GCL) thickness according to age- and sex-adjusted normative values, in the presence or absence of visual field defects. **Results**: Ten patients (mean age: 22.2 ± 3.6 years) were assessed. They all presented with symptoms typical of N_2_O-induced neuropathy or myelopathy and elevated blood methylmalonic acid and total homocysteine levels, which are markers of vitamin B12 inactivation. Three patients showed decreased RNFL and GCL thickness. The cumulative exposure to N_2_O was higher (3220 ± 2801 kg vs. 165 ± 158 kg) and the consumption duration was longer (36 ± 32 vs. 8 ± 8 months) in these three patients compared to those without optic nerve involvement. **Conclusions**: This preliminary case series suggests that massive and prolonged N_2_O exposure may be associated with subclinical optic neuropathy, possibly mediated by vitamin B12 inactivation. Given the growing prevalence of recreational N_2_O use, ophthalmological evaluation including OCT could be considered in patients with heavy or prolonged use and neurological symptoms. These findings are hypothesis-generating and require confirmation in larger prospective studies.

## 1. Introduction

The recent increase in recreational nitrous oxide (N_2_O) use, also known as “laughing gas”, has been associated with multiple neurological disorders affecting both the peripheral and central nervous systems [1,2,3]. Although the neurological manifestations of N_2_O abuse have been extensively characterized, its ophthalmological complications remain unexplored [4,5,6]. N_2_O oxidizes the cobalt ion of cobalamin, inactivating vitamin B12, which is a cofactor for methionine synthase and methylmalonyl-CoA mutase, resulting in impaired myelin synthesis and in the accumulation of neurotoxic metabolites such as homocysteine and methylmalonic acid (MMA). Vitamin B12 deficiency, known to cause neurological, hematological and psychiatric manifestations, rarely manifests as optic neuropathy, which is typically painless, progressive, and reversible when treated early. It could be assumed that N_2_O abuse could similarly affect the optic nerve function through vitamin B12 inactivation, as observed in classic etiologies of vitamin B12 deficiency (e.g., pernicious anemia, restrictive diets, or malabsorption).

The aim of this study was to investigate this assumption by performing comprehensive ophthalmological assessments in consecutive patients hospitalized in the department of neurology of our hospital for laughing gas-related symptoms.

## 2. Materials and Methods

### 2.1. Study Design and Participants

A retrospective, monocentric, observational case series was conducted, including all patients hospitalized for confirmed N_2_O-related neurological symptoms in the neurology department of Avicenne Hospital (Bobigny, France) between January 2019 and May 2025. Forty eligible patients were contacted, and 10 consented to participate in the ophthalmological evaluation, which was performed between January 2019 and July 2025. The study adhered to the tenets of the Declaration of Helsinki. Participants gave non-opposition agreement.

### 2.2. Data Collection

Patients’ sex, age, employment status, medical history, use of other substances, N_2_O exposure, neurological symptoms, and complementary findings, including magnetic resonance imaging (MRI), electroneuromyography (ENMG), and blood tests, were retrieved from the medical records and patient interviews conducted by the ophthalmologist. Total N_2_O exposure was estimated in kilograms (kg) based on self-reported consumption. One cartridge (equivalent to one balloon) was assumed to contain 8 g of N_2_O [7]. Unless specified otherwise by the patient, a gas cylinder was considered to contain approximately 800 g of N_2_O. Continuous variables are summarized as mean ± standard deviation. Owing to the small sample size, no formal statistical comparison was undertaken, and no *p*-values are reported; all between-group differences, including in the duration and cumulative dose of N_2_O exposure, are descriptive only.

### 2.3. Neurological Assessment

The diagnosis of N_2_O-related myelopathy or peripheral neuropathy was based on the combination of N_2_O abuse and characteristic clinical manifestations, along with elevated blood MMA and total homocysteine levels, ENMG abnormalities, and, in some cases, spinal cord lesions on MRI. As the clinical presentation could mimic other conditions, differential diagnoses and other vitamin deficiencies were systematically ruled out. Vitamin B12 supplementation was generally initiated at admission to the neurology department, in most cases around or shortly after the blood sampling used to measure vitamin B12, homocysteine and methylmalonic acid; the ophthalmological examination was subsequently performed at a variable interval after supplementation had begun (Table 1).

### 2.4. Ophthalmological Assessment

Ophthalmological evaluation included the best-corrected visual acuity (BCVA), intraocular pressure (IOP), anterior and posterior segment slit-lamp biomicroscopy, spectral-domain optical coherence tomography (OCT) (Spectralis OCT, Heidelberg, Germany, and/or DRI Triton OCT, Topcon, Japan), and visual field testing (24-2 Humphrey Field Analyzer, SITA standard, Carl Zeiss Meditec, Germany or Goldmann kinetic perimetry, Haag-Streit, Bern, Germany). All OCT examinations and visual field testing were performed by experienced orthoptists or trained ophthalmologists. Automatic segmentation was generated by the device software and independently reviewed by two ophthalmologists (J.A. and H.T.-V.); only scans of adequate quality with correct segmentation were retained, and the two ophthalmologists’ classification of RNFL and GCL as normal or abnormal was concordant for all ten patients. Spectralis SD-OCT (Spectral Domain OCT) was used as the primary device for RNFL analysis, and the DRI Triton was used as a confirmatory second device when available. As absolute retinal thickness values differ between platforms, measurements were not pooled across devices; each value was interpreted against the corresponding device’s own age- and sex-adjusted normative database, and, when both devices were available, only concordant results were retained. Suspected optic neuropathy was defined as an abnormal total thickness of the Retinal Nerve Fiber Layer (RNFL) and Ganglion Cell Layer (GCL), according to age- and sex-adjusted normative values provided by the OCT device, with or without associated visual field defects. To limit misclassification, abnormal thickness was required to be bilateral and concordant between the two OCT platforms when both were available, and to follow a sectoral pattern compatible with toxic or nutritional optic neuropathy (predominant temporal thinning or diffuse thinning involving the papillomacular projection) rather than an isolated single-sector or unilateral change.

Abnormal RNFL or GCL thickness was classified as moderate when the color code displayed by the OCT device was yellow (corresponding to a probability of normality <5%) and as severe when the color code was red (corresponding to a probability of normality <1%).

## 3. Results

Ten patients hospitalized for N_2_O-related myelopathy or peripheral neuropathy were included and underwent an ophthalmological examination. Their demographics and clinical characteristics are summarized in Table 1.

The mean age was 22.2 ± 3.6 years (range: 18–28), with an equal representation of women and men. Eight out of the 10 patients were unemployed. Half of the patients reported the concomitant use of other substances, including alcohol, cannabis, or both. The mean duration of N_2_O use before hospitalization was 16 ± 21 months, and the mean cumulative N_2_O exposure was 1082 ± 1985 kg. All patients presented with symptomatic neuropathy or myelopathy typical of N_2_O-related neurological damage, elevated blood MMA and total homocysteine levels, and abnormal spinal MRI findings in 7 cases (patients 3 to 9). Vitamin B12 deficiency was found in 3 patients (patients 3, 5 and 8). The mean time between the hospitalization in the neurology department and the ophthalmological examination was 15 ± 16 months (range: 0–36). At the time of their first ophthalmological evaluation, four patients (patients 3, 4, 7 and 8) had not discontinued using N_2_O.

Of the 10 patients included, the development of N_2_O-induced optic neuropathy was suspected in three patients (patients 1, 2 and 3). These cases are detailed below. These three patients were all women, and none reported concomitant alcohol or cannabis use. Two of them had a history of sleeve gastrectomy. They all also had a longer history of N_2_O use than patients without optic neuropathy (36 ± 32 months vs. 8 ± 8 months, respectively), and a higher cumulative N_2_O exposure (3220 ± 2801 kg vs. 165 ± 158 kg), with patients 1 and 2 reporting a massive consumption of 5256 kg and 4380 kg, respectively. Furthermore, patients with suspected N_2_O-induced optic neuropathy had higher blood MMA and total homocysteine levels at the time of diagnosis (9.4 ± 11.2 µmol/L vs. 4.8 ± 2.7 µmol/L and 90.7 ± 41.2 µmol/L vs. 65.9 ± 32.6 µmol/L, respectively). The mean RNFL thickness in these three patients was 82.1 ± 12.9 µm, compared to 109.5 ± 6.3 µm in patients without optic neuropathy. All the patients without optic neuropathy had a normal RNFL thickness according to the age- and sex-adjusted normative values provided by the OCT device.

### 3.1. Patient 1

Patient 1 was a 23-year-old woman with a history of heavy N_2_O consumption, estimated at around 5256 kg over six years (equivalent to three gas cylinders per day). She did not report any other toxic ingestion, including alcohol, and her only past medical history was sleeve gastrectomy that she had undergone three years earlier.

In the months following sleeve gastrectomy, she lost 30 kg, and while she had already been using N_2_O for several years, she gradually developed neurological symptoms, including limb weakness, sensory deficits in the lower extremities, urinary incontinence, memory loss, and attention disorders. Neurological examination and ENMG were highly suggestive of N_2_O-induced myeloneuropathy. Spinal MRI performed four months after discontinuing N_2_O use was normal, while brain MRI revealed incidental lateral sinus thrombosis that was treated with anticoagulants. At the time of diagnosis, serum vitamin B12 and total homocysteine levels were 439 ng/L (reference range: 187–883 ng/L) and 47.8 µmol/L (reference range: <15.0 µmol/L), respectively, supporting N_2_O-induced toxicity.

An ophthalmological examination was performed six months after N_2_O discontinuation. The patient reported no visual complaints. The BCVA and IOP were normal in both eyes. Slit-lamp examination, including anterior and posterior segment assessment, revealed no abnormalities. Spectralis OCT showed a moderate RNFL thinning with moderate inferotemporal atrophy in both eyes (total RNFL thickness: 82 µm in the right eye (OD) and 85 µm in the left eye (OS)). Triton OCT confirmed a moderate bilateral inferotemporal RNFL thinning (88 µm OD and 89 µm OS). GCL measurement showed a moderate thinning in both eyes (56 µm OD and 56 µm OS). The 24-2 Humphrey visual field testing was unreliable due to fixation losses and an excessive number of false-positive and false-negative responses.

### 3.2. Patient 2

Patient 2 was a 21-year-old woman with an estimated total N_2_O consumption of 4380 kg over two years (one 6 L carboy per day for 2 years, with occasional massive acute inhalation from N_2_O tanks). She reported no other toxic ingestion, including alcohol, and had no relevant medical history. She presented with ataxia, sensory disorders including paresthesia, motor deficiency, urinary urgency and incontinence. Neurological examination and ENMG were highly suggestive of N_2_O-induced myeloneuropathy. Spinal MRI was normal at the time of diagnosis. Serum vitamin B12 level was within normal range (439 ng/L, reference range: 187–883 ng/L). However, total homocysteine and MMA levels were elevated at the time of diagnosis, at 94.2 µmol/L (reference range: <15.0 µmol/L) and 17.3 µmol/L (reference range: <0.4 µmol/L), respectively, supporting N_2_O toxicity. Two ophthalmological evaluations were performed one and four years after discontinuation of N_2_O use. The BCVA and IOP were normal in both eyes. Slit-lamp examinations, including anterior and posterior segment assessment, did not show any particularity. The first SD-OCT performed using the Spectralis one year after N_2_O discontinuation showed a reduced RNFL thickness in both eyes (OD: 91 µm and OS: 96 µm) with a moderate temporal thinning. Follow-up Spectralis OCT performed four years after N_2_O discontinuation showed normal RNFL values (OD: 97 µm and OS: 100 µm). Triton OCT showed a similar initial RNFL pattern (OD: 102 µm and OS: 102 µm), with moderate temporal atrophy, and subsequent measurements were within the normal range (OD: 103 µm and OS: 108 µm). The GCL thickness was not measured. Humphrey visual field testing performed at the second examination was normal for both eyes.

### 3.3. Patient 3

Patient 3 was a 28-year-old woman with an estimated N_2_O consumption of 25 kg (approximately 60 balloons per weekend for one year). She reported smoking but no other toxic consumption, including alcohol, and had a history of sleeve gastrectomy performed seven years prior to her hospitalization, which had led to a 100-kg weight loss over this period.

After one year of N_2_O use, she rapidly developed progressive sensory deficits, ataxia and general weakness. Spinal MRI revealed posterior cord T2 hyperintensity consistent with myeloneuropathy. Blood tests revealed macrocytic anemia (hemoglobin level: 9 g/dL), markedly reduced serum vitamin B12 levels (86 ng/L; reference range: 187–883 ng/L), and elevated total homocysteine (130.0 µmol/L; reference range: <15.0 µmol/L) and MMA (1.5 µmol/L; reference range: <0.4 µmol/L) levels. An ophthalmological evaluation was performed during hospitalization because the patient complained of bilateral vision loss. The BCVA and IOP were normal. Slit-lamp examination, including anterior and posterior segment assessment, was unremarkable. Spectralis SD-OCT performed at the time of diagnosis showed a bilateral reduced RNFL thickness (OD: 71 µm and OS: 72 µm), with severe inferior, superior and temporal atrophy. Follow-up Spectralis OCT performed three years after N_2_O discontinuation showed stable RNFL values in both eyes (OD: 68 µm and OS: 71 µm), with persistent inferior, superior and temporal atrophy. Triton OCT performed at the same time revealed a similar bilateral RNFL thinning (OD: 73 µm and OS: 75 µm). GCL analysis showed a moderate bilateral thinning (OD: 57 µm and OS: 56 µm). Visual field testing with Goldmann perimetry revealed severe bilateral constriction (Figure 1). The symptoms remained stable after three years of vitamin B12 supplementation.

## 4. Discussion

This case series reported 10 patients who underwent an ophthalmological examination following a diagnosis of N_2_O-related neurological disease. To our knowledge, this is the first description of the effects of N_2_O consumption on the optic nerve. OCT revealed evidence of optic nerve involvement in three patients with a history of massive and prolonged N_2_O use. These patients had higher cumulative and longer exposure to N_2_O compared to those without optic nerve abnormalities.

Our cohort is comparable to those described in previous studies, consisting mainly of young adults with similar socioeconomic backgrounds, often socially isolated, unemployed, and showing patterns of polysubstance use [8]. This demographic context likely reflects both the accessibility of N_2_O as a recreational drug and the psychosocial vulnerability associated with its chronic abuse. These characteristics are consistent with the largest series published to date: in a multicenter UK cohort of 119 patients with N_2_O-induced myeloneuropathy, paraesthesia was the presenting complaint in most cases, the mid-cervical cord was the region most often involved on MRI, and the weekly number of canisters consumed correlated with blood methylmalonic acid levels [8]. Comparable increases have since been reported in other European centers [9], and an individual patient data meta-analysis pooling medical and recreational exposures confirmed that neurological involvement dominates the clinical picture [10]. Our cohort therefore appears representative of the population currently described worldwide.

The biological mechanism proposed to explain N_2_O-related neurotoxicity is that N_2_O could induce a functional vitamin B12 deficiency by inactivating it. N_2_O oxidizes the cobalt ion within cobalamin (from Co^+^ to Co^3+^), inducing a functional vitamin B12 deficiency by making cobalamin unable to accept methyl groups. As a result, the levels of methylcobalamin, a cofactor necessary for methionine synthase, decrease, which ultimately impacts DNA synthesis and methylation processes [11,12]. This biochemical profile explains why serum vitamin B12 is frequently normal in N_2_O users despite established functional deficiency: in a systematic review of laboratory abnormalities in recreational users, elevated homocysteine and methylmalonic acid were far more consistent markers than serum cobalamin itself [13]. This supports our decision to base the diagnosis of functional vitamin B12 deficiency on these metabolites rather than on serum B12 levels.

Classic neurological damage includes both axonal and demyelinating peripheral neuropathies, as well as myeloneuropathies [14,15,16]. Grzych et al. have recently observed that heavy N_2_O consumers show various electrophysiological manifestations [17]. N_2_O-induced neuropathy typically presents as length-dependent sensorimotor axonal neuropathy characterized by profound motor axonal loss mainly affecting the lower limbs, but subacute demyelinating patterns have also been observed [18]. Interestingly, patients with demyelinating patterns showed less severe hyperhomocysteinemia and lower MMA levels.

The optic nerve involvement observed in our three patients could result from N_2_O-induced damage, through both demyelinating and axonal degeneration. Indeed, other causes of vitamin B12 deficiency have been associated with optic nerve degeneration, and more rarely, with optic neuritis [6,19]. Temporal RNFL thinning in the context of vitamin B12 deficiency is well documented [20]. Koca et al. have shown that vitamin B12 deficiency anemia leads to a decrease in temporal RNFL thickness [21]. One hypothesis is that vitamin B12 deficiency could primarily affect the cells consuming the most energy, such as those located in the papillomacular bundle. The preferential temporal RNFL involvement observed in our cohort reflects the findings in classic vitamin B12 deficiency-related optic neuropathy and other mitochondrial optic neuropathies (e.g., Leber hereditary optic neuropathy, ethambutol toxicity). This pattern could reflect the high mitochondrial density, low myelin reserve, and high metabolic demand of the papillomacular bundle fibers, making them particularly vulnerable to oxidative and metabolic stress [22,23]. The structural signature of toxic and nutritional optic neuropathies on OCT has been refined in recent years. In a series of five patients with toxic or nutritional optic neuropathy, diffuse early loss of the retinal ganglion cell layer was observed with relative preservation of the RNFL, indicating that the ganglion cell bodies may be affected before measurable axonal thinning appears [24]. This dissociation is directly relevant to the present study, in which the diagnosis rested primarily on RNFL analysis, and reinforces the possibility that early optic nerve involvement was underestimated in our cohort. Beyond global thickness values, both inter-eye asymmetry and sectoral analysis provided additional information. The three patients with suspected optic neuropathy showed markedly symmetrical RNFL values between the two eyes (inter-eye differences of 3, 5 and 1 µm for patients 1, 2 and 3, respectively), and this symmetry persisted over follow-up. Such symmetry is characteristic of toxic, nutritional and metabolic optic neuropathies and argues against compressive, ischemic or inflammatory causes, which are usually asymmetrical. Sectoral analysis showed that all three patients had a temporal component of thinning: inferotemporal in patients 1 and 2, and temporal associated with superior and inferior involvement in patient 3, who had the most advanced disease. The consistent temporal involvement supports preferential vulnerability of the papillomacular bundle and provides diagnostic specificity that global thickness values alone do not convey. Conversely, none of the patients without suspected optic neuropathy showed isolated sectoral abnormalities.

It is also noteworthy that during patient 1′s hospitalization, incidental lateral sinus thrombosis was identified on MRI. Although a direct causal relationship cannot be firmly established, cases of cerebral venous thrombosis have been reported in association with chronic N_2_O exposure [25,26], probably due to high plasma levels of homocysteine, a known risk factor for cardiovascular diseases. This finding could therefore be an additional factor contributing to the patient’s optic neuropathy, potentially mediated by transient intracranial hypertension.

Our study sample was small, but we observed that the three patients presenting with optic neuropathy had higher cumulative and longer N_2_O exposure, likely reflecting higher homocysteine and MMA levels. In the literature, high homocysteine levels have been associated with recent N_2_O consumption, whereas high MMA levels could reflect clinical severity [27]. Patients 1 and 2 with optic neuropathy were the only ones with a total N_2_O consumption exceeding 1000 kg, suggesting a possible association with the dose. In patient 2, the RNFL values shifted from the abnormal to the normal range of the device’s age- and sex-adjusted normative database between the first and last examinations. However, the absolute change was small (≈2 µm in the right eye according to the Spectralis) and lies within the reported test–retest reproducibility of RNFL measurements (approximately ±5 µm). No clinically meaningful structural recovery can therefore be claimed, and this change is most likely attributable to measurement variability rather than to true axonal regeneration or reinnervation. No relationship could be established between the presence of optic neuropathy and the serum vitamin B12 levels at the time of diagnosis, as these measurements were largely uninterpretable. In many cases, the patients had already received vitamin B12 supplementation prior to blood sampling due to the presence of symptoms typical of N_2_O toxicity, which could have biased the results.

Patients 1 and 2 presented with subclinical optic neuropathy (structural OCT anomalies without visual complaints or visual field defects), while patient 3 presented with clinical optic neuropathy (OCT anomalies and visual field reduction). Patient 3 was the only patient with visual symptoms and neurological impairments related not only to N_2_O abuse, but also to nutritional deficiencies, including vitamin B12 deficiency, exacerbated by the weight loss of 100 kg over 7 years following sleeve gastrectomy. This finding suggests that N_2_O-related optic neuropathies could not reach the threshold required to cause visual field defects unless they co-occur with other neuropathy etiologies. Two of the three patients with suspected optic neuropathy had undergone sleeve gastrectomy, a recognized cause of vitamin B12 malabsorption and nutritional optic neuropathy, and one (patient 1) also presented with an incidental lateral sinus thrombosis. These coexisting factors are important confounders. Rather than being a sole and sufficient cause, N_2_O more plausibly acted as an aggravating factor superimposed on a pre-existing nutritional vulnerability, and, in patient 1, a concomitant venous/prothrombotic component, that together lowered the threshold for optic nerve dysfunction.

Our observational case series was a preliminary study, the main limitation of which is its small sample size. Only 10 out of the 40 patients contacted underwent ophthalmological examinations, which considerably limited the generalizability of our findings. Only 10 of the 40 eligible patients (25%) agreed to undergo ophthalmological evaluation, which introduces substantial selection bias, as participants may differ systematically from non-participants. Because participants may have differed from non-participants in either direction, patients with visual symptoms may have been more likely to attend, whereas the most severely affected or socially precarious patients were frequently lost to follow-up; both overestimation and underestimation of the true prevalence of optic nerve involvement are possible, and the direction of this bias cannot be determined. For these reasons, our findings cannot be generalized to the wider population of N_2_O users and should be regarded as preliminary and hypothesis-generating, intended to justify larger prospective studies rather than to establish a definitive association. This challenge was also evident in the medical records, as many patients often did not attend neurological consultations and were lost to follow-up. Furthermore, patients were contacted retrospectively, except for patients 3, 7 and 8, with variable time intervals between their hospitalization and the ophthalmological examination, which could potentially have confounded the effect of time on optic nerve function and recovery. Additionally, toxic N_2_O consumption was self-reported and sometimes estimated using standardized volumes, which could not accurately reflect the actual intake. Because cumulative N_2_O exposure was self-reported and back-calculated from standardized cartridge and cylinder volumes, the absolute values are approximations and are likely subject to substantial recall and conversion error. The exact figures (e.g., 3220 vs. 165 kg) should therefore not be over-interpreted; they should be read as indicating a difference in order of magnitude of exposure between groups rather than precise quantities.

Given the very small number of patients (*n* = 10, three with suspected optic nerve involvement), no inferential statistical analysis was performed; all comparisons are descriptive and should be regarded as hypothesis-generating rather than confirmatory.

The structural definition of optic neuropathy has intrinsic limits: device-based normative comparisons are subject to test–retest variability, and mild RNFL thinning is not specific to toxic optic neuropathy. Although all three suspected cases showed bilateral thinning with a temporal component, functional confirmation was incomplete, as visual fields were unreliable or normal in two of them. Visual evoked potentials would have strengthened the diagnosis by providing objective functional evidence of optic nerve dysfunction; unfortunately, they were not available in this retrospective series and could not be obtained a posteriori because several patients were lost to follow-up. Prospective inclusion of VEP is warranted in future studies. The diagnosis of optic neuropathy was based primarily on RNFL thickness rather than on the macular ganglion cell layer/complex (GCL). We recognize that the ganglion cell bodies, which have a high metabolic demand, are typically affected earliest in toxic and nutritional optic neuropathies, and that GCL analysis is more sensitive than RNFL for detecting early damage. Our choice was dictated by data quality: a large proportion of examinations were acquired on the Spectralis platform, on which reliable and reproducible a posteriori ganglion cell segmentation was difficult to obtain consistently across devices and follow-up visits, whereas RNFL measurements from both platforms were reliable. Two OCT platforms were used, which is a further limitation. Absolute retinal thickness values are not interchangeable between devices because of differences in light source, scan-circle diameter, segmentation algorithms and normative databases. To limit this effect, measurements were never pooled across devices: each value was interpreted only against the normative database of the device that generated it, longitudinal comparisons for a given patient were made within a single platform, and Spectralis was used as the primary device throughout. When both devices were available, they were qualitatively concordant regarding the presence and sectoral distribution of thinning. Nonetheless, the use of two platforms may have introduced some heterogeneity, and a single-device protocol should be preferred in future prospective studies. As RNFL tends to underestimate toxic and metabolic optic neuropathy until a relatively advanced stage, the true prevalence of optic nerve involvement in our cohort may have been underestimated, and future prospective studies should use standardized GCL protocols as the primary structural marker.

It is also possible that the use of other substances was not disclosed, which could be a confounding factor. Finally, the diagnosis of optic neuropathy was based on the analysis of OCT findings. Among the three patients with optic neuropathy, patients 1 and 2 presented only with RNFL and GCL thinning, as the visual field results were either uninterpretable (patient 1) or normal (patient 2). Only patient 3 showed an abnormal visual field, and these findings warrant particular caution. The severe, roughly concentric bilateral constriction observed is atypical for nutritional or metabolic optic neuropathy, which characteristically produces central or cecocentral scotomas with a relatively preserved peripheral field, reflecting preferential involvement of the papillomacular bundle [28,29]. Furthermore, there was a marked structure–function mismatch, as the degree of functional constriction was disproportionate to the moderate RNFL and GCL thinning measured on OCT. This discrepancy, together with the non-physiological field pattern, raises the possibility of a functional (non-organic) visual overlay, which is not uncommon in this young, socially vulnerable population and may coexist with genuine organic damage. In addition, Goldmann kinetic perimetry, although appropriate for less cooperative patients, is examiner-dependent and may accentuate apparent constriction. Taken together, we interpret these findings as indicating a genuine but mild-to-moderate organic optic neuropathy, as supported by the OCT changes, while the severe concentric field constriction is most likely largely functional in origin. Objective functional testing such as visual evoked potentials, together with formal structure–function correlation, would be required to confirm this interpretation and should be incorporated in future studies.

## 5. Conclusions

This preliminary case series provides imaging findings compatible with a subclinical optic neuropathy in a subset of patients with massive and prolonged N_2_O exposure, with an apparent relationship with the cumulative dose and duration of use. Although no patient experienced significant visual loss, the OCT abnormalities were accompanied by elevated biological markers of vitamin B12 inactivation. These observations are preliminary and hypothesis-generating and require confirmation in larger prospective studies using standardized ganglion cell analysis and functional testing. In the meantime, given the increasing prevalence of recreational N_2_O use, ophthalmological evaluation including OCT could be considered in patients with heavy or prolonged N_2_O use and neurological symptoms.

## Figures and Tables

**Figure 1:**
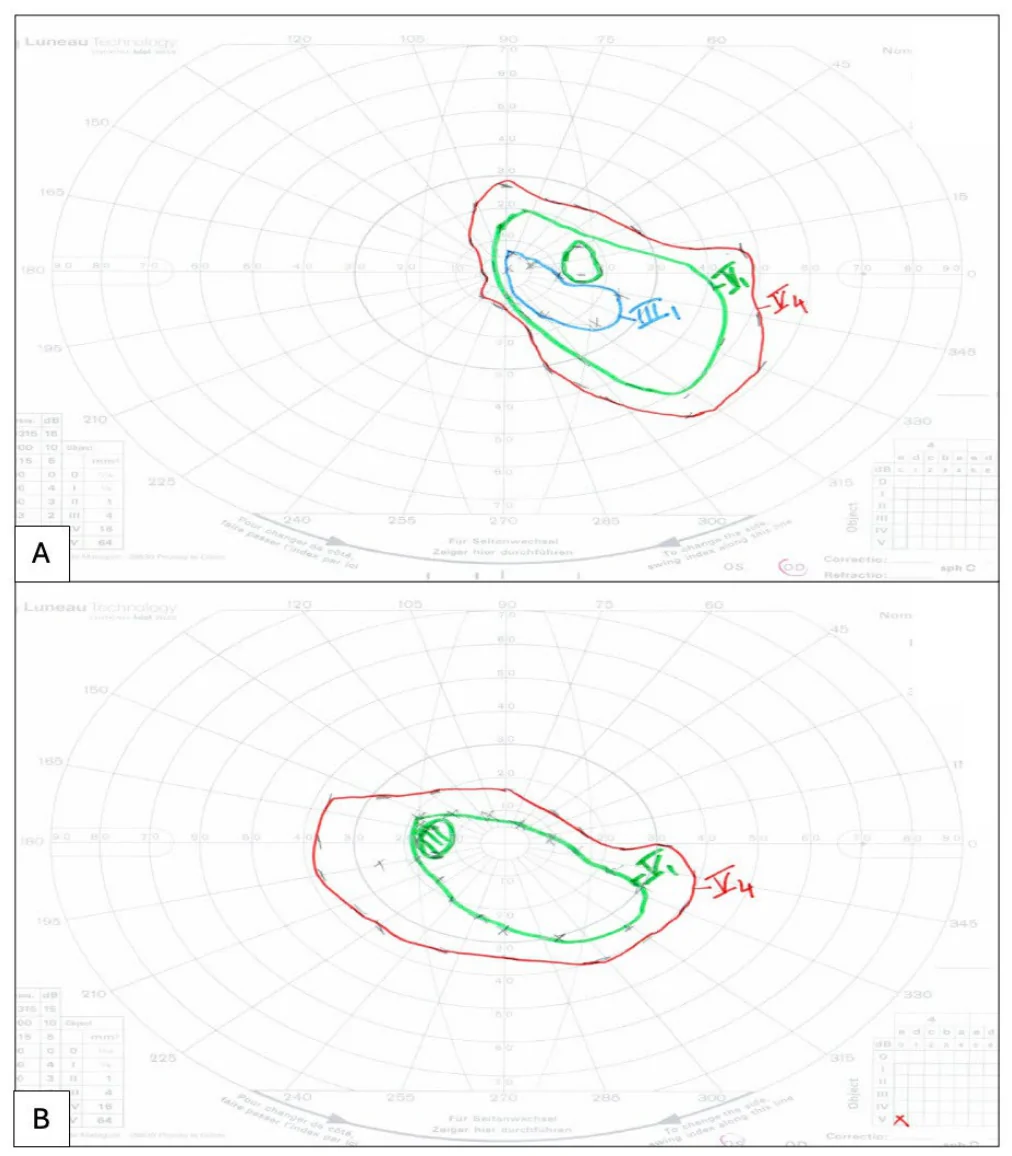
Goldmann visual fields showing bilateral constriction in patient 3 with suspected N_2_O-induced optic neuropathy ((**A**): right eye; (**B**): left eye). Roman numerals indicate stimulus size (III = 4 mm^2^, V = 64 mm^2^) and Arabic numerals indicate stimulus intensity (1–4, 4 being the brightest). Isopters: V4 (red), V1 (green), III1 (blue).

**Table 1:** Demographics and clinical characteristics of 10 patients hospitalized for N_2_O-induced neuropathy.

	Patient 1	Patient 2	Patient 3	Patient 4	Patient 5	Patient 6	Patient 7	Patient 8	Patient 9	Patient 10
Age (years)	23	21	28	27	21	19	26	20	18	19
Sex	Female	Female	Female	Male	Female	Male	Female	Male	Male	Male
Employment status	Unemployed	Student	Unemployed	Unemployed	Unemployed	Unemployed	Unemployed	Delivery driver	Unemployed	Unemployed
Use of other substances	—	—	—	—	Alcohol	Alcohol and cannabis	Alcohol	Cannabis	Cannabis	Cannabis
Medical history	Sleeve gastrectomy	—	Sleeve gastrectomy	—	—	—	—	—	—	—
Type of neurological involvement (neuropathy, myelopathy or mixed)	Mixed	Mixed	Myelopathy	Mixed	Mixed	Myelopathy	Mixed	Mixed	Mixed	Mixed
Time between the diagnosis and the first ophthalmological examination	6 months	2 months	0	3 years	3 years	3 years	1 month	6 months	3 months	2 years
Discontinuation of N_2_O at the time of ophthalmological evaluation	Yes	Yes	No	No	Yes	Yes	No	No	Yes	Yes
Duration of N_2_O use	6 years	2 years	1 year	4 months	2 years	1 year	1 week	6 months	1 week	1 year
Cumulative N_2_O exposure (kg)	5256	4380	25	48	350	416	28	146	23	146
Blood methylmalonic acid level (µmol/L, reference range: <0.4 µmol/L)	NA	17.3	1.5	6.8	6.7	3.8	1.4	7.3	4.9	2.7
Homocysteinemia (µmol/L, reference range: <15.0 µmol/L)	47.8	94.2	130.0	38.8	21.3	70.1	45.0	113.0	78.3	95.0
Vitamin B12 deficiency	No	No	Yes	No	No	Yes	No	Yes	No	No
Suspected optic neuropathy	Yes	Yes	Yes	No	No	No	No	No	No	No
RNFL thickness (µm) (OD/OS) *	82/85	91/96	71/72	104/104	117/120	107/104	107/108	102/107	118/119	105/111

N_2_O: nitrous oxide; OD: right eye; OS: left eye; RNFL: retinal nerve fiber layer; NA: not available. *: RNFL thickness measured by Spectralis OCT.

## Data Availability

The data supporting the findings of this study are available from the corresponding author upon reasonable request. The data are not publicly available due to privacy restrictions.

## Abbreviations

The following abbreviations are used in this manuscript:

BCVA	Best-corrected visual acuity
ENMG	Electroneuromyography
GCL	Ganglion cell layer
IOP	Intraocular pressure
MMA	Methylmalonic acid
MRI	Magnetic resonance imaging
N_2_O	Nitrous oxide
NA	Not available
OCT	Optical coherence tomography
RNFL	Retinal nerve fiber layer
SD-OCT	Spectral-domain optical coherence tomography
